# Anti-inflammatory and immuno-modulatory studies on LC-MS characterised methanol extract of *Gentiana kurroo* Royle

**DOI:** 10.1186/s12906-017-1593-7

**Published:** 2017-01-28

**Authors:** Khan Mubashir, Bashir A. Ganai, Khalid Ghazanfar, Seema Akbar, Bilal Rah, Mudasir Tantry, Akbar Masood

**Affiliations:** 1 School of Medicine, Goba Referral Hospital, Madda Walabu University, Bale Robe, Ethiopia; 2 Centre for Research and Development, University of Kashmir, Srinagar, 190 006 India; 3 Indian Institute of Integrative Medicine (CSIR), Canal road, Jammu, India; 4 Regional Research Institute of Unani Medicine, Kashmir University Campus, Srinagar, 190006 India; 5 Department of Biochemistry, University of Kashmir, Srinagar, 190 006 India

**Keywords:** *Gentiana kurroo*, Delayed type hypersensitivity, IL-6, Inflammation, TNF-α, NO

## Abstract

**Background:**

In ayurvedic traditional medicine *Gentiana kurroo* Royle (family; *Gentianaceae*) is used to treat several metabolic diseases. This plant is rich in various compounds belonging to flavonoids and glycosides. Till now little work has been carried out on immunomodulatory and anti-inflammatory potential of this plant. This study confirms the presence of bioactive compounds and evaluates the anti-inflammatory and immunomodulatory effect of this plant.

**Methods:**

To carry out this work, the methanol extract was investigated in different doses using in vivo and in vitro models. In vivo study involved haemagglutination titre and DTH methods, and in vitro study was done using splenocyte proliferation assay and LPS stimulated macrophage culture. TNF-α, IL-6 and NO were assayed using ELISA kit methods, while NF-*κ*B was evaluated by western blotting. LC-ESI-MS/MS was used for the characterization of the methanol extract.

**Results:**

The results showed suppression of both humoral and cell mediated immunity in vivo. This effect was also observed by inhibition of B and T cell proliferation in splenocyte proliferation assay. TNF-α, IL-6 and NO concentrations were also less in extract treated macrophage cultures. The NF-*κ*B expression was also lowered in treated macrophages as compared to untreated macrophages. All these observations were found to be dose dependent. LC-MS characterization of this extract showed the presence of known compounds which are glycosides, alkaloids and flavonoids in nature.

**Conclusion:**

The methanol extract of this plant was found to be rich in glycoside, alkaloid and flavonoid compounds. These compounds are probably responsible for the suppression of immune response and anti-inflammatory activity. The extract as such and identified bioactive compounds can be useful for the treatment of inflammatory disorders.

## Background

Inflammation is an essential protective process preserving the integrity of organisms against physical, chemical, and infective insults. Inflammatory diseases majorly develop by altering the response of T-cells and causing irregularity in immune system function starting with the migration of leukocytes, primarily neutrophils [1]. Further, during inflammation the pro-inflammatory molecules like Nitric oxide (NO) and TNF-α are produced by macrophages and the inhibition of NO synthase and TNF-α reverses inflammatory symptoms [2]. This inflammatory response is transduced by a variety of signalling pathways. Nuclear factor kappa B (NF-*κ*B) is one of the essential signalling molecules acting as a transcription factor for the expression of iNOS, TNF-α and IL-6 genes by binding to their promoter regions [3, 4]. Many synthetic drugs like glucocorticoids are used for the treatment of inflammatory disorders by immune system modulation, but none of them is without serious adverse effects and are thus highly unsafe for use in humans. The different synthetic drugs used against inflammation are reported to produce drug related toxicities, adverse reactions and iatrogenic effects complicating the treatment process [5, 6]. Hence, anti-inflammatory treatment has observed a shift from synthetic to herbal products. These herbal products with fewer side effects are mainly the primary and secondary metabolites [7]. Such herbal drugs with the capacity to inhibit cellular and humoral immune responses can have useful applications in some immune-mediated disorders including autoimmune diseases [8].

In this perspective the current study was carried out on *Gentiana kurroo* Royle used as an effective drug in ayurveda for the treatment of several metabolic diseases [9]. Further the flower tops of this plant has been shown to possess the anti-inflammatory potential [10]. In our earlier studies different extracts of this plant were screened for anti-inflammatory and immuno-modulatory potential and methanol extract was found to show the maximum potential [11]. So in the current study this extract was extensively assesssed for its effect on immune response to explore its protective mechanism against inflammation.

## Methods

### Collection, identification and selection of herbal substance

*Gentiana kurroo* Royle locally known as Nilkanth (English name- Indian Gentian) was procured from sub-alpine region in Dachigam and identified in the Centre of Plant Taxonomy (COPT), Dept. of Botany, University of Kashmir. The specimen is retained in the herbarium of COPT vide voucher no.1804-KASH. Whole plant material was selected for the study.

### Preparation of extracts

The plant material was cleaned, cut into small pieces and dried under a shade. It was then pulverized into coarse powder in an electric blender, weighed (~180 g dry weight) and extracted successively using petroleum ether, ethyl acetate, methanol and water respectively by soxhlet extraction each for a period of 96 h. The solvents were allowed to evaporate in a rotary evaporator at 40–45 °C and the extracts obtained were stored in a refrigerator at 4 °C. The yields of the petroleum ether, ethyl acetate, methanol and aqueous extracts were 5.6, 4.3, 5.3, and 4.2% (*w/w*), respectively.

### Animals

Male Balb/C mice 8–10 weeks old and weighing 18–22 g, were used for the immuno-modulatory study. The animals were housed under standard laboratory conditions with a temperature of (25 ± 2)°C, relative humidity of (55 ± 10)%, 12/12 h light–dark cycles and fed with standard pellet diet (Lipton India Ltd.) and water was given *ad libitum*.

### Experimental protocols

All experimental protocols and the number of animals used for the experimental work were duly approved by the Institutional Animals Ethics Committee (IAEC); of Indian Institute of Integrative Medicine (CSIR), Canal Road Jammu (CPCSEA registration No. 67/CPCSEA/99). The experimental work on animals was carried out as per the guidelines set by committee for the purpose of control and supervision of experiments on animals (CPCSEA).

### Preliminary toxicity study

Five male Balb/C mice 8–10 weeks old were taken. Each animal was tested with the methanol extract at an oral dosage of 2000 mg/kg bw as per OECD guidelines. The mice were observed for 24 h for any signs of toxicity including change in behaviour or death.

### Humoral antibody response

The mice were divided into 6 groups, each group consisting of 5 animals. Mice in group I (control) were given 1% tween 20, 0.2 mL/mice for 14 days. Mice in group II-V were given the extract at a dose range of 25, 50, 100, 200 mg/kg bw respectively for 14 days. Mice in group VI were given cyclophosphamide (50 mg/kg) on day 1 and continued for 14 days. All drugs were given orally. The animals were immunised by injecting 200 μL of 5 × 10^9^ SRBC’s/mL intraperitonially (i.p) on day 1. Blood samples were collected in microliter tubes from individual animals in all the groups by retro-orbital vein puncture on day 7 and day 14. The blood samples were centrifuged and the serum was separated. Then haemoglutination primary and secondary titres were performed [12, 13].

### Delayed type hypersensitivity

This method was carried out to determine the effect of extracts on the cell-mediated immunity. Animals were divided into six groups of 5 each. Group I served as sensitized control, as in humoral antibody titre. Mice in group II-V were administered methanol extract at a dose range of 25, 50, 100, 200 mg/kg bw respectively after SRBC’s sensitization (i.p), once daily for 14 days. Cyclophosphamide (50 mg/kg bw) was administered as standard T-cell suppressor (group VI). The mice were challenged on day 14 by injecting 0.02 mL of SRBC’s antigen subcutaneous into the right hind footpad, whereas left hind footpad served as control [14, 15]. The footpad thickness was measured with micrometre calliper (pitch 0.01 mm) after 24 and 48 h of SRBC’s challenge.

### Ex-vivo TNF-α assay

The male Balb/C mice dosed for 14 days with variable concentrations (25–200 mg/kg) of methanol extract were analysed for serum TNF-α. Blood (0.5 mL) was taken on the 14th day by retro-orbital puncture from the mice used for the study of DTH reaction. Blood was kept on standing for 2 h at room temperature (24 °C). After clotting of blood, the tubes were centrifuged at 8000 rpm for 10 min, at 4 °C. The serum so collected was evaluated for the measurement of TNF-α using mouse TNF-α ELISA kit (R&D systems, USA), as per the instructions of the manufacturer.

### Collection of peritoneal macrophages and nitrite assay

Male Balb/C mice were i.p. injected 4% sodium thioglycollate media 1 mL per mice. Under aseptic conditions mice were euthanized after 24 h and injected 10 mL of RPMI 1640 medium in peritoneal cavity. After 5 min, the medium was taken out and centrifuged at 1800× g for 10 min at 4 °C. The cell pellet was resuspended in RPMI 1640 medium. The macrophages (3× 10^6^) were cultured in 96 well micro-plate in the presence of LPS (1 μg/mL), β-Methasone (0.001 μg/mL) and variable doses of methanol extract (25–200 μg/mL). The plates were incubated at 37 °C in a humid saturated atmosphere containing 5% CO_2_ for 24 h. Supernatants were collected after centrifugation and stored at −80 °C for determination of cytokines. The supernatants were used for nitrite assay using Griess reagent. For the NO_2_ assay (nitrite content), 100 μl of the culture media was incubated with 100 μL Griess reagent (0.1% naphthyl-ethylenediamide and 1% sulphanilamide in 2.5% phosphoric acid solution) at room temperature for 10 min in 96 well micro-plate [16]. Optical density was measured at 540 nm using ELISA plate reader. The nitrite concentration was determined by extrapolation from a sodium nitrite (NaNO_2_) standard curve and the results expressed in μM.

### IL-6 and TNF-α measurements

The harvested supernatants from cultured peritoneal macrophages were used for the measurement of pro-inflammatory cytokines (IL-6 and TNF-α) using mouse IL-6 and TNF-α ELISA kits (R&D systems, USA), as per the instructions of the manufacturer.

### Lymphocyte proliferation studies on mice splenocytes

Spleen collected under aseptic conditions in IRPMI, was minced using a pair of scissors and passed though a fine steel mesh to obtain a homogenous cell suspension and the erythrocytes were lysed with ammonium chloride (0.8% *w/v*). After centrifugation (380 × g at 4 °C for 10 min), the pelleted cells were washed three times with PBS and resuspended in complete medium [RPMI 1640 supplemented with 12 mM HEPES (pH 7.1), 0.05 mM 2-mercaptoethanol, 100 IU/mL penicillin, 100 μg/mL streptomycin and 10% FBS]. The cell number was counted with a haemocytometer by the trypan blue dye exclusion technique. Cell viability exceeded 95% [17].

To evaluate the effect of methanol extract on the proliferation of splenic lymphocytes, the spleen cell suspension (2 × 10^6^ cells/mL) was pipetted into 96 well plates (200 μl/well) and cultured at 37 °C for 72 h in a humid saturated atmosphere containing 5% CO_2_ in the presence of Con-A (5 μg/mL), LPS (1 μg/mL) and variable doses of methanol extract (25–200 μg/mL). β-methasone (BMS) was used as standard drug at a concentration of 0.001 μg/mL. After 72 h, 20 μL of MTT (5 mg/mL) was added to each well and plates were incubated for 4 h. The plates were centrifuged (1400 × g, 5 min) and the untransformed MTT was removed carefully by pippeting. To each well, 100 μl of DMSO working solution (192 μl DMSO with 8 μl 1 M HCl) was added and the absorbance was evaluated in ELISA reader at 570 nm after 15 min. Reduction of MTT indicated the splenocyte proliferation.

### Western blot analysis

Peritoneal macrophages collected from male Balb/C (0.5 × 10^6^ cells) were incubated overnight and exposed to different concentrations of methanol extract (25–100 μg/mL) with DMSO as vehicle and β-methasone (0.001 μg/mL) as positive control. LPS (1 μg/mL) was also added to all wells except the vehicle, thus having two vehicles - DMSO as vehicle (unstimulated), vehicle + LPS (stimulated) so as to have a better understanding of the results. Cells were accordingly rinsed with PBS, trypsinized and collected after 24 h incubation and were lysed with lysis buffer (HEPES 1 mM/L, KCl 60 mM/L, NP-40 0.3%, EDTA 1 mM/L, DTT 1 mM/L, Sodium orthovandate 1 mM/L, PMSF 0.1 mM/L, cocktail protease inhibitor). The cell extractions were centrifuged at 12,000 rpm for 10 min at 4 °C. Supernatant was collected for protein quantification. Protein concentration was determined by the standard Bradford method [18]. Equal amount (20 μg) of protein from each sample was subjected to SDS-PAGE (12%) and proteins were transferred to membrane (Millipore), blocked with 5% (*w/v*) non-fat milk in PBS containing 0.1% Tween-20 and probed with anti-NF-кB (p65) and anti-β-actin for 3 h at room temperature overnight at 4 °C and subsequently washed, probed with species specific secondary antibodies coupled to horseradish peroxidase. Immuno-reactive proteins were detected by enhanced chemiluminescence plus (Amersham).

### Liquid chomatography–tandem mass spectrometry (LC–ESI-MSMS)

LC–MS equipment (LC–MS QqQ-6410B Agilent Technologies) comprised a chomatographic system (1260 Infinity Agilent Technologies) coupled with an Agilent Triple Quad mass spectrometer fitted with an ESI source. MS conditions were the following: MS range 100–1200 Da, MSn spectra were obtained using both positive and negative modes, nebulizer gas 45 Psi, gas temperature 325 °C, capillary voltage 4000 V.

HPLC analysis was carried out by an Agilent 1260 infinity series. A Chomolith RP-18e column (4.6 mm ID, 50 mm length) (Merck) was used. Mobile phase consisted of (A) aqueous formic acid (0.1%) and (B) methanol. Gradient condition was; 0–8 min, linear gradient from 12 to 25% of B; 8–12 min, isocratic conditions at 25% of B; 12–16 min, linear gradient from 25 to 40% of B; 16–40 min, linear gradient from 40 to 50% of B, 40–50 min, linear gradient from 50 to 100% of B. Flow rate: 1 mL/min.

### Statistical analysis

Data is expressed as Mean ± SD and statistical analysis was carried out employing the one-way ANOVA followed by Post Hoc Tukey’s HSD test and Bonferroni multiple comparison test using SPSS 16 software. *p* values < 0.05 are being taken as statistically significant.

## Results

### Preliminary toxicity study

The methanol extract was found to be non-toxic at a dose of 2000 mg/kg bw.

### Haemagglutination antibody titre

**Fig. 1:**
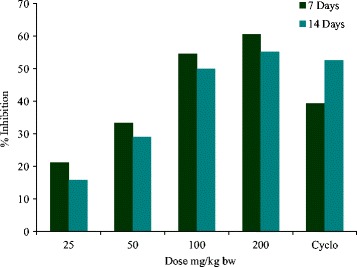
Graph showing %age inhibition of primary and secondary humoral response in Balb/C mice by methanol extract (25–200 mg/kg). Cyclophosphamide (50 mg/kg) was taken as standard drug

In the current study as is evident from Table 1, the methanol extract showed increased activity with the increase in dose. The effect was found to be more in primary titre than in the secondary titre. At a dose of 200 mg/kg the methanol extract had maximum effect on HA titre and also more than cyclophosphamide. The inhibition at this dose by methanol extract in primary and secondary humoral response observed was 66.21 and 55.26% respectively (Fig. 1). Cyclophosphamide treated group showed 39.39 and 52.63% inhibitory response in the primary & secondary titres. Statistical analysis showed significance at *p* < 0.05, of the results obtained between the control and the treated groups.

**Table 1:** Effect of methanol extract on SRBC specific humoral immune response in Balb/C mice. (mean ± S.E) (*n* = 5)

Humoral response			
Treatment	Dose (mg/kg)	Primary titre	Secondary titre
Control	SRBC	6.6 ± 0.24^a^	7.6 ± 0.24^a^
I	25	5.2 ± 0.2^ab^	6.4 ± 0.24^b^
II	50	4.4 ± 0.24^bc^	5.4 ± 0.24^b^
III	100	3 ± 0.45^cd^	3.8 ± 0.37^c^
IV	200	2.6 ± 0.24^d^	3.4 ± 0.24^c^
Cyclophosphamide	50	4 ± 0.45^bcd^	3.6 ± 0.24^c^

Values along the same column with different superscripts are statistically significant to each other using Tukey’s HSD test (*p* < 0.05)

### Delayed type hypersensitivity

**Fig. 2:**
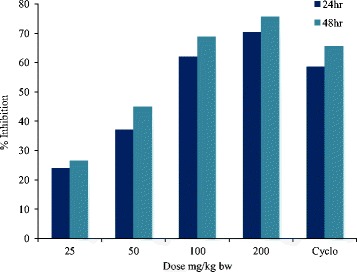
Graph showing 24 and 48 h %age inhibition of cell mediated response in Balb/C mice by methanol extract (25–200 mg/kg). Cyclophosphamide (50 mg/kg) was taken as standard drug

DTH was carried out to study the effect of different doses of methanol extract on non-specific cellular immune response. Results obtained showed dose dependent effect in cellular immune response (Table 2). In control group edema formation was high, but was found to be less in treated groups. In positive control group the edema formation was less than control group but methanol extract treated group at a dose of 200 mg/kg had least edema formation with an inhibition of 70.33 & 75.66% in cellular response after 24 & 48 h respectively, while in cyclophosphamide treated group (50 mg/kg bw) the suppression in cellular immune response was 58.52 & 65.61% as compared to control group (Fig. 2). The results obtained were found to be significant in relation with control group at *p* < 0.05.

**Table 2:** Effect of methanol extract on SRBC specific cellular immune response in Balb/C mice. (mean ± S.E) (*n* = 5)

DTH ASSAY			
Treatment	Dose (mg/kg)	24 h paw thickness (mm)	48 h paw thickness (mm)
Control	SRBC	0.728 ± 0.01^a^	0.378 ± 0.007^a^
I	25	0.554 ± 0.034^b^	0.278 ± 0.018^b^
II	50	0.458 ± 0.034^c^	0.208 ± 0.011^c^
III	100	0.276 ± 0.009^d^	0.118 ± 0.011^d^
IV	200	0.216 ± 0.01^d^	0.092 ± 0.002^d^
Cyclophosphamide	50	0.302 ± 0.01^d^	0.13 ± 0.01^d^

Values along the same column with different superscripts are statistically significant to each other using Tukey’s HSD test (*p* < 0.05)

### Ex-vivo TNF-α assay

**Fig. 3:**
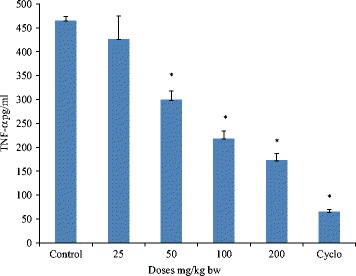
Effect of methanol extract on serum TNF-α concentration in male Balb/C mice. The results in graph include control group, methanol extract treated groups (25–200 mg/kg) and positive control group treated with cyclophosphamide (50 mg/kg). Values are mean ± SD (*n* = 5); *p < 0.05 (control vs. treated groups); one-way ANOVA followed by Bonferroni multiple comparison test

The results indicated a decrease in serum TNF-α levels of treated groups as compared to control in a dose dependent manner. The group administered with methanol extract at a dose range of 200 mg/kg significantly (*p* < 0.05) decreased serum TNF-α (174.96 pg/mL) as compared to control (468.09 pg/mL). Cyclophosphamide treated group significantly (*p* < 0.05) decreased the TNF-α in serum (Fig. 3).

### Effect of methanol extract on NO production in macrophages

**Fig. 4:**
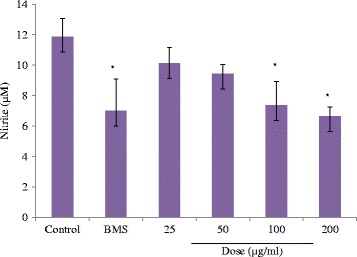
Effect of methanol extract on the nitrite content in mice peritoneal macrophages. Values are means ± SD; *p < 0.05 (control vs. treated groups); one-way ANOVA followed by Bonferroni multiple comparison test. All tests were carried out in triplets and repeated twice

Since NO is also known as a pro-inflammatory mediator in different acute and chronic inflammatory diseases, we addressed whether methanol extract modulates the NO production from macrophages stimulated by LPS. The extract inhibited the NO production in a dose dependent manner at a dose range of 25–200 μg/mL. The maximum inhibition was observed at 200 μg/mL dose compared to control. Increase in the dose of methanol extract indicated a significant inhibition in the production of nitrite content (Fig. 4). BMS also showed a significant inhibition (*p* < 0.05) in nitrite content compared to control.

### Effect of methanol extract on the TNF-α and IL-6 release in mice peritoneal macrophages stimulated with LPS

**Fig. 5:**
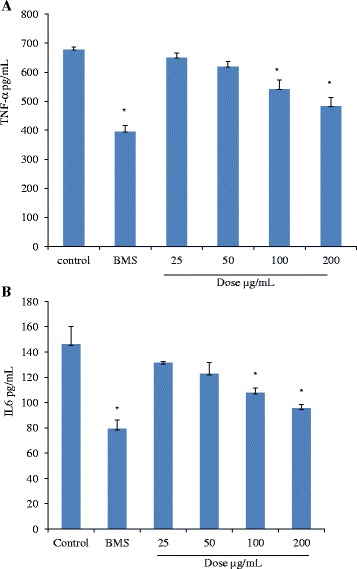
**a** Effect of methanol extract on TNF-α release in mice peritoneal macrophages. Values are mean ± SD., *p < 0.05 (control vs. treated groups); one-way ANOVA followed by Bonferroni multiple comparison test. All tests were carried out in triplets and repeated twice. **b** Effect of methanol extract on IL-6 release in mice peritoneal macrophages. Values are mean ± SD., *p < 0.05 (control vs. treated groups); one-way ANOVA followed by Bonferroni multiple comparison test. All tests were carried out in triplets and repeated twice

Results indicated that the extract inhibited the release of these two cytokines from macrophages in a dose dependent manner. At a dose of 200 μg/mL, inhibition in TNF-α (Fig. 5a) and IL-6 (Fig. 5b) was found to be most significant (*p* < 0.05). BMS also registered a significant inhibition (*p* < 0.05) in the release of TNF-α and IL-6 from LPS stimulated macrophages compared to control.

### Effect of methanol extract on splenocyte proliferation assay

**Fig. 6:**
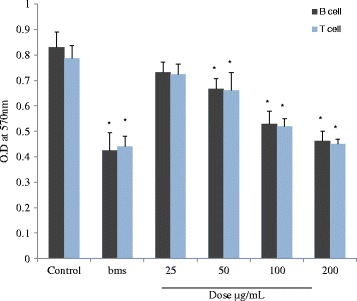
Effect of methanol extract on ConA and LPS-stimulated splenocyte cell proliferation. Values are mean ± SD showing OD at 570 nm, *p < 0.05 (control vs. treated groups); one-way ANOVA followed by Bonferroni multiple comparison test. All tests were carried out in triplets and repeated twice

The results observed were found to show suppression of both T and B-cell proliferation of splenocytes at doses (25–200 μg/mL) in a dose dependent manner compared to control. Maximum observation was registered at a dose of 200 μg/mL (Fig. 6). The extract indicated more inhibition of T-cell than the B-cell; however the difference was not so high. β-methasone (0.001 μg/mL) showed significant inhibition (*p* < 0.05) of both T and B-cell proliferation compared to control.

### Effect of methanol extract on NF-kappa B expression in mice peritoneal macrophages stimulated with LPS

**Fig. 7:**
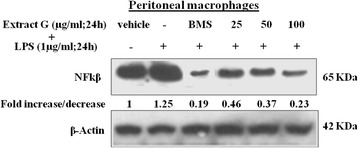
NF- КB (p65) expression in mice peritoneal macrophages. Extract treated cultures were found to show decreased expression of NF-kappa B (p65) as compared to stimulated and unstimulated vehicle. β-actin was used as loading control

In presence of LPS (stimulated) increased concentrations of NF- кB (p65) were registered in protein extracts of peritoneal macrophages after 24 h as compared to vehicle (unstimulated). The results indicated reduced expression of NF-kappa B (p65) in presence of the methanol extract. The methanol extract was used in a dose range of 25–100 μg/mL and showed dose-dependent inhibition in the expression of NF-kappa B (p65). At a dose of 100 μg/mL maximum inhibition was observed up to 0.23 fold decrease in its expression (Fig. 7). BMS used as standard drug showed significant decrease in the expression of NF-kappa B (p65).

### LC–ESI-MS/MS analysis

**Fig. 8:**
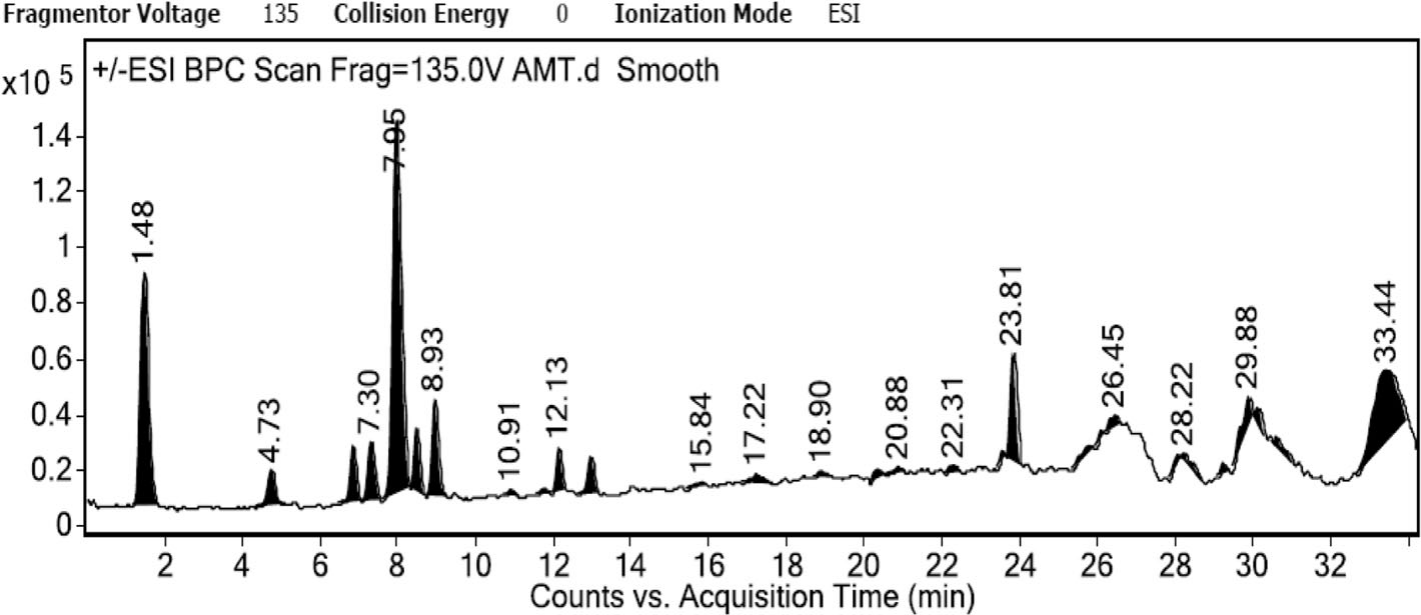
Total Ion Chomatogram (TIC) of the methanol extract

**Fig. 9:**
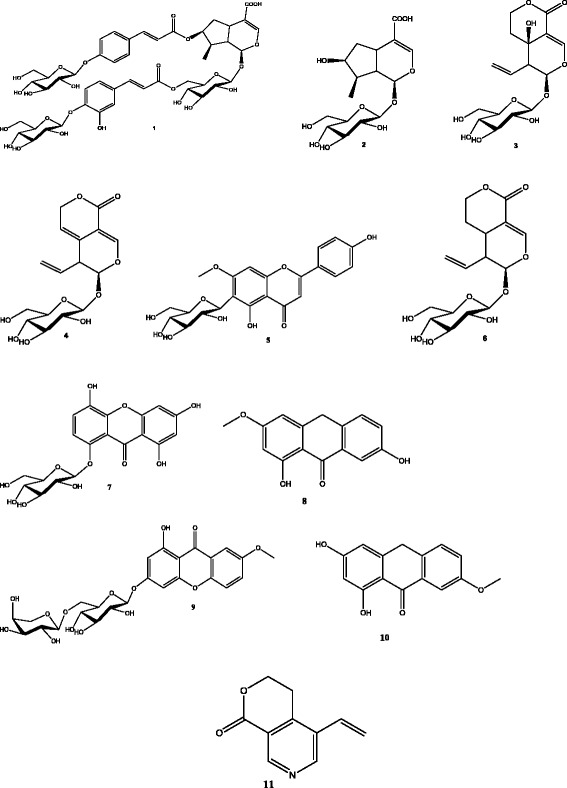
Structures of the various identified compounds in the methanol extract. **1** 4″-O-β-D-glucosyl-6′-O-(4-O-β-D-glucosylcaffeoyl)-linearoside. **2** Loganic acid, **3** Swertiamarin, **4** Gentiopicroside, **5** Swertisin, **6** Sweroside, **7** Norswertianolin, **8** Gentisin, **9** Gentioside, **10** Isogentisin, **11** Gentianine

The methanol extract was analyzed for the presence of different compounds by liquid chomatography tandem mass spectrometry (LC-ESI-MS/MS). The total ion MS chomatogram (TIC) as shown in Fig. 8 showed a number of peaks corresponding to different chemical constituents present in the extract. The major peaks were further analysed under both positive and negative ESI-MS modes. Further the molecular ion peaks along with the MS fragmentation pattern obtained were compared with the molecular ion peaks of different compounds reported in the literature for the identification of the compounds [19–22]. The compounds identified are given in the Table 3 showing their actual mass (m/z), mass obtained in both ESI^+^/ESI^−^ modes, retention time and area sum%. The compounds in the ESI^+^ mode [M + Na]^+^ at m/z 399.00, 397.00, 379.00, 279.00 coincides with loganic acid (m/z = 376.13), swertiamarin (m/z = 374.12), gentiopicroside (m/z = 356.11), gentisin (m/z = 256.07) respectively. In ESI^+^ [M + K]^+^ mass spectra mode at m/z 214.90 single compound gentianine (m/z = 175.06) was identified. The compounds sweroside (m/z = 358.12) and norswertianolin (m/z = 422.07) were identified in the ESI^+^ [M + H]^+^ mass spectra mode at m/z 359.10 and 423.00, while in ESI^−^ [M – H]^−^ mass spectra mode at m/z 1007.10, 444.90, 551.20 and 255.10 match with the measured masses of 4′′-O-β-D-glucosyl-6′-O-(4-O-β-D- glucosyl caffeoyl) linearoside (m/z = 1008.31), swertisin (m/z = 446.12), gentioside (m/z = 552.14) and isogentisin (m/z = 256.07) (Fig. 9).

**Table 3:** Identified compounds in the methanol extract by LC-ESI-MS/MS

S.No.	Compound	Actual mass (*m/z*)	ESI^+^ (*m/z*)	ESI^−^ (*m/z*)	RT (min)	Area sum%
1.	4″-O-β-D-glucosyl-6′-O-(4-O-β-D-glucosylcaffeoyl)-linearoside	1008.31		1007.10 [M - H]^−^	1.48	20.42
2.	Loganic acid	376.13	399.00 [M + Na]^+^		6.84	3.58
3.	Swertiamarin	374.12	397.00 [M + Na]^+^		7.3	4.25
4.	Gentiopicroside	356.11	379.00 [M + Na]^+^		7.95	32.66
5.	Swertisin	446.12		444.90 [M - H]^−^	7.95	32.66
6.	Sweroside	358.12	359.10 [M + H]^+^		8.48	3.58
7.	Norswertianolin	422.07	423.00 [M + H]^+^		12.13	2.41
8.	Gentisin	256.07	279.00 [M + Na]^+^		23.81	6.43
9.	Gentioside	552.14		551.20 [M - H]^−^	28.22	0.13
10.	Isogentisin	256.07		255.10 [M - H]^−^	29.88	1.52
11.	Gentianine	175.06	214.90 [M + K]^+^		33.44	17.85

*RT* Retention time

## Discussion

Inflammation is a protective process that is essential for the preservation of the integrity of the organism in the event of physical, chemical and infectious damage. Often, it is found that inflammatory response to severe lesions erroneously damages normal tissue [23]. It is well established that activated immunocytes are involved in inflammation process, particularly macrophages, which play a crucial role in specific and nonspecific immune responses during inflammation [24]. So the methanol fraction was used to demonstrate the effect on the immune system using HA titre and DTH method for determining its effect on humoral and cell mediated immune responses respectively. The methanol fraction was observed to have an immunosuppressive effect by inhibiting the antibody formation and the cellular immune response in a dose dependent manner. One of the major pro-inflammatory cytokine is TNF-α which is produced by different immune cells and its rate of production and hence its concentration in blood demonstrates the extent of inflammatory response [25]. Blood samples were taken from the treated and untreated groups of animals used in the HA titre and DTH models. The results indicated marked difference in the concentration of serum TNF-α between the treated and untreated groups. Further the serum TNF-α concentration in serum of treated animals showed decrease with increase in dose of the methanol extract and hence the dose dependent effect. During the progression of inflammation, the most known important mediators produced by macrophages include NO, prostanoids, TNF-$\alpha$, and Interleukins [26, 27]. Among these inflammatory cytokines, TNF-$\alpha$, IL-1$\beta$, and IL-6 are considered the most crucial mediators for the inflammatory process, mediating immunity and activating macrophages [28, 29]. Lipopolysaccharide (LPS)-stimulated macrophages using Raw264.7 cells is a well-established in vitro inflammation model [29, 30]. This model was used for investigating suppressive effect of methanol extract on the release of inflammatory mediators-NO, TNF-α and IL-6 by LPS activated macrophages. The extract inhibited the production of these mediators in a dose dependent manner from LPS stimulated macrophages. These results infer that the contribution of methanol extract in anti-inflammatory activity might be by inhibition of the production of these pro-inflammatory mediators from immune cells. The effect of methanol extract on the proliferation of B-cells and T-cells in response to LPS and ConA respectively, was investigated using MTT assay. The methanol extract was found to significantly inhibit the proliferation of both cells in a dose dependent manner. The extract indicated more inhibition of T-cell than the B-cell; however the difference was not so high. These results are consistent with above results where the methanol extract inhibited the humoral and cell mediated immune response. It is well known that B-cells produce antibodies in response to the antigens while T-cells help in framing the cellular response against the antigens. Because NF-$\kappa$B acts as transcription factor and thus causes induction of iNOS, TNF-$\alpha$, and IL-6 genes by binding to their promoter regions [3, 4, 31–33]. So it was essential to investigate its expression while studying the production of these cytokines. The expression of NF-кB (p65) in mice peritoneal macrophages stimulated with LPS was reduced in presence of different concentrations of the methanol extract. So the reduced production of the pro-inflammatory mediators observed above may be attributed to inhibition in the expression of NF-кB (p65) and hence the effect against the inflammation.

The plant secondary metabolites - Iridoids and flavonoids are reported to have anti-inflammatory potential [34, 35]. Various compounds belonging to Iridoids and flavonoids were found to be present in methanol extract. Among them loganic acid, swertiamarin and gentianine are reported to possess the anti-inflammatory potential [36–38]. The anti-inflammatory potential of methanol extract is probably due to these compounds.

## Conclusion

Our research results demonstrate that *G. kurroo* possess anti- inflammatory activity. This activity was mediated by suppression of immune system- where both the immune cells as well as the cytokines were found to be inhibited. The plant extract as such and the identified bioactive compounds like the loganic acid, swertiamarin and gentianine may be used as natural anti-inflammatory compounds.

## Abbreviations

%: Percent
BMS: Betamethasone
bw: Body weight
C: Degree centigrade
DMSO: Dimethyl sulfoxide
ELISA: Enzyme linked immunosorbent assay
g: Gram
h: Hour
HCl: Hydrochloric acid
IL-6: Interleukin 6
iNOS: Induced nitric oxide synthase
kg: Kilogram
LPS: Lipopolysaccharide
mg: Milligram
min: Minutes
mL: Millilitre
mM: Millimolar
NF-$\kappa$B: Nuclear factor kappa B
NO: Nitric oxide
PBS: Phosphate buffered saline
pg: Pictogram
SD: Standard deviation
SRBC: Sheep red blood cells
TNF-α: Tumor necrosis factor alpha
μg: Microgram
μl: Microlitre
